# CD34 and Bcl-2 as predictors for the efficacy of neoadjuvant chemotherapy in cervical cancer

**DOI:** 10.1007/s00404-020-05921-8

**Published:** 2021-01-03

**Authors:** Yun Lin, Zhi Li, Mubiao Liu, Haiyan Ye, Jianhui He, Jianguo Chen

**Affiliations:** 1 Department of Gynecology, Guangdong Provincial People’s Hospital, Guangdong Academy of Medical Sciences, Guangzhou, China; 2 Shantou University Medical College, Shantou, China; 3 Department of Pathology, Guangdong Provincial People’s Hospital, Guangdong Academy of Medical Sciences, Guangzhou, China

**Keywords:** CD34, Bcl-2, Neoadjuvant chemotherapy, Cervical cancer

## Abstract

**Background:**

Successful neoadjuvant chemotherapy (NACT) could improve the surgical resection rate and radical curability of patients with cervical cancer, but only a subset of patients benefits. Therefore, identifying predictive biomarkers are urgently needed. The aim of this study was to evaluate the predictive value of CD34 and Bcl-2 in the NACT effectiveness of cervical cancer.

**Methods:**

Sixty-seven patients with locally advanced cervical cancer (FIGO stages IB3, IIA2 or IIB) were classified into two groups based on effective (*n* = 48) and ineffective (*n* = 19) response to NACT. Immunohistochemistry was employed to identify CD34 and Bcl-2 expression before and after NACT. We analyzed the associations between the pre-NACT expression of these two biomarkers and the response of NACT. The expression of these two biomarkers before and after NACT was also assessed and compared.

**Results:**

More patients were CD34 positive expression before NACT in effective group compared to ineffective group (*p* = 0.005). However, no statistically significant difference in Bcl-2 expression before NACT were found between two groups (*p* = 0.084). In NACT effective group, the expression of both CD34 and Bcl-2 after NACT are down-regulated (*p* < 0.001 and *p* < 0.001, respectively), while there are no statistical differences between the pre- and post-NACT expression of CD34 and Bcl-2 in NACT ineffective group (*p* = 0.453 and *p* = 0.317, respectively).

**Conclusion:**

The positive CD34 expression before NACT may serve as a predictive biomarker for NACT of cervical cancer, but the pre-NACT expression of Bcl-2 is not an independent predictor. The down-regulated expression of these two indicators after NACT may indicate effective NACT.

## Introduction

With an estimated 570,000 new cases and 311,000 deaths in 2018 worldwide, cervical cancer ranks as the fourth most frequently diagnosed cancer in women [1]. For decades, radical hysterectomy and concurrent chemoradiation have been applied to treat cervical cancer of FIGO stages IB3 to IIB. However, the overall and recurrence-free survival rate were not significantly improved [2]. Previous study found that the recurrence-free survival was significantly longer in patients who achieved an overall optimal response to NACT than in those who did not, suggesting that effective NACT may be a good indicator to prognose the survival of the patients [3]. However, not all patients have a response to NACT. Insensitive to NACT may result in poorer prognosis and ultimately patient mortality. Therefore, identifying the biomarkers to assist the prediction of prognosis and efficacy of NACT is needed.

Both angiogenesis and apoptosis play essential roles in the growth and metastasis of cervical cancer. Exploring the biomarkers involved in these two pathways may reveal the mechanism of NACT for cervical cancer. CD34, a transmembrane glycoprotein expressed in capillary endothelial cells, is a useful angiogenesis marker reflecting the grade of microvascular modeling in cervical cancer [4–6]. Bcl-2 plays an important role in the intrinsic apoptotic pathway of malignant cancer and has been shown to interfere with chemotherapy [7]. Preferentially, the expression of CD34 and Bcl-2 were proved to be the predictors of neoadjuvant chemotherapy in urothelial bladder cancer [8] and breast cancer [9, 10]. However, up until now, less study has been dedicated to clarify the association between CD34 or Bcl-2 expression and the response to NACT in cervical cancer.

Therefore, to determine the utility of CD34 and Bcl-2 expression as predictive biomarkers, we investigate the relationship between the expression of two biomarkers before NACT and the efficacy of NACT in cervical cancer. Besides, we also sought to analyze the variation of CD34 and Bcl-2 regarding different NACT response.

## Material and methods

### Patients and tissue sample preparation

We enrolled 67 patients diagnosed with cervical cancer who underwent NACT at Guangdong Provincial People's Hospital between January to December 2018. None of these patients underwent any anti-cancer therapies before NACT. Any subject with other malignant diseases or serious complications was excluded. The histopathology of tissue samples was confirmed by pathologists, and all included patients were squamous cell carcinoma (SCC). Magnetic resonance imaging of pelvic and computer tomography of thorax and abdomen were performed to all patients before and after NACT. The stage of tumor was determined according to the International Federation of Gynecology and Obstetrics (FIGO 2018) classification guideline based on clinical examination, colposcopy and imaging.

Three cycles platinum-paclitaxel NACT followed radical hysterectomy with pelvic lymphadenectomy were performed to all included patients. The NACT regimen consisted of cisplatin (70–75 mg/m^2^) combined with paclitaxel (135-175 mg/m^2^) were given to patients via intravenous infusion every 21 days. Patients were classified into two groups based on the response to NACT. Clinical examination, colposcopy and imaging were used to evaluate the response to NACT, based upon the changes in the local cervical lesion. The size of the cervical tumor was assessed as the product of anteroposterior diameter and transverse diameter. According to the World Health Organization (WHO) RECIST-criteria (Response Evaluation Criteria In Solid Tumors), the tumor response was classified into the complete response (CR), partial response (PR), stable disease (SD), and progressive disease (PD) [11]. CR is defined as complete disappearance of local tumor, PR as at least 30% reduction, PD as at least 20% increase of lesion or appearance of a new lesion, SD as neither sufficient shrinkage to qualify for PR, nor sufficient increase to qualify for PD. The subjects with CR and PR were classified as NACT effective group, while SD and PD were classified as NACT ineffective group. Tumor tissue samples of cervical cancer were obtained by punch biopsy before and after NACT.

### Immunohistochemical staining

**Fig. 1:**
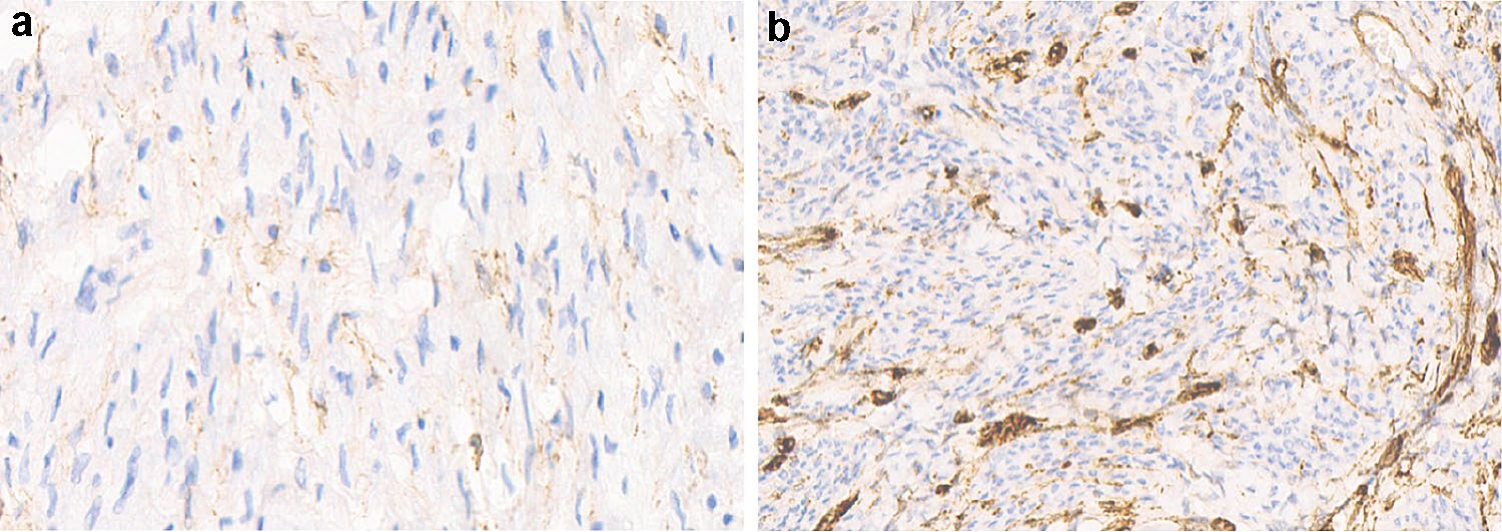
Immunohistochemical staining of CD34 in cervical cancer. **a** Negative staining. **b** Positive staining. Magnification, ×400

**Fig. 2:**
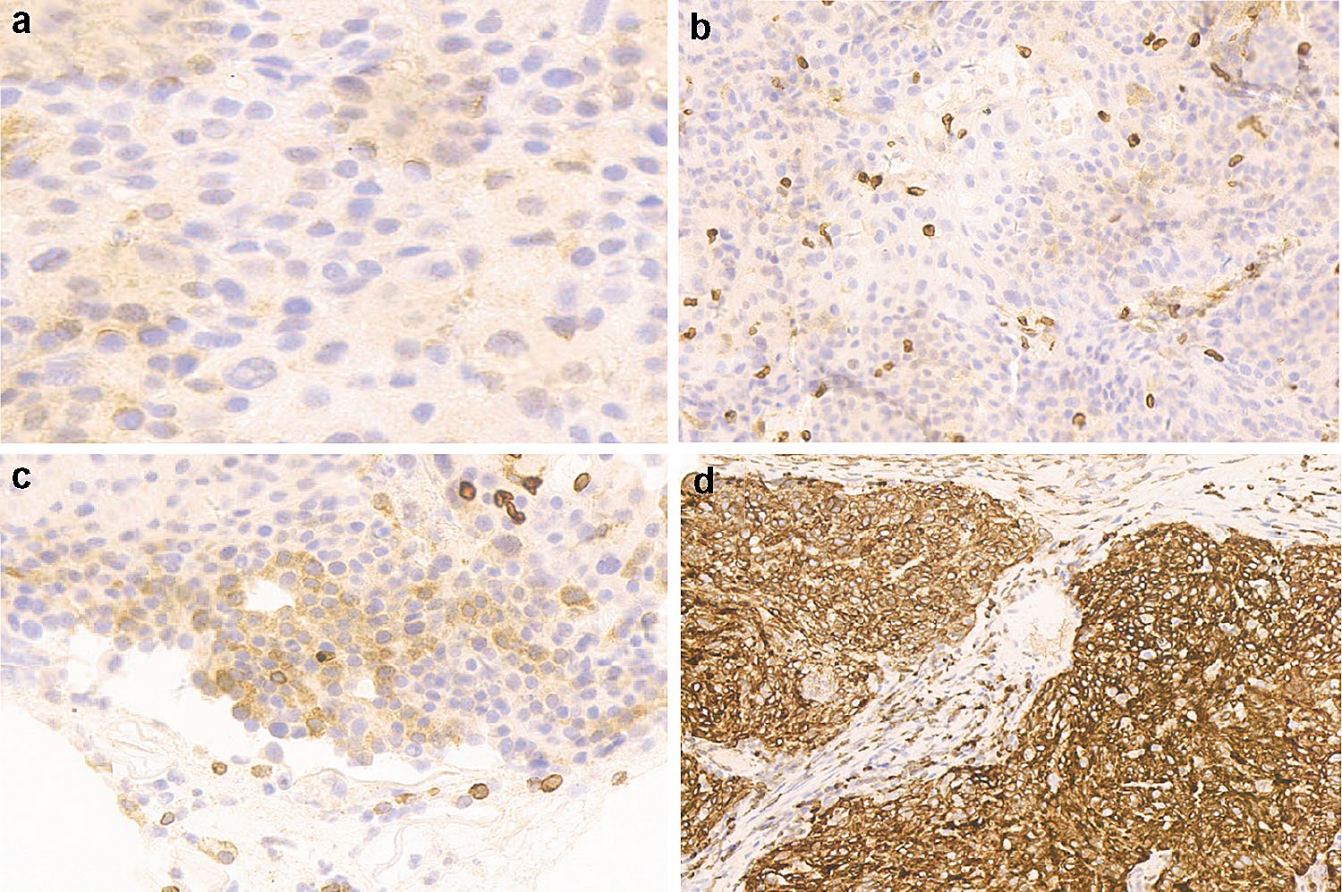
Immunohistochemical staining of Bcl-2 in cervical cancer. **a** Negative staining. **b** Weak staining. **c** Moderate staining. **d** Intense staining Magnification, ×400

For immunohistochemistry, 4-μm sections placed on gelatin-coated slides were cut from formalin-fixed, paraffin-embedded tissues, and were sequentially dried, dewaxed, re-hydrated and rinsed. The sections were immersed in 0.3% H_2_O_2_ for 10 min to block endogenous peroxidase activity and boiled in citrate buffer (pH 6.0) for 10 min in a pressure cooker for antigen retrieval. Then the sections were incubated respectively with rabbit antibodies against CD34 (1:200 dilution; Fine Test, China) and Bcl-2 (1:200 dilution; OmnimAbs, USA) overnight at 4 °C. The antibodies were diluted by primary antibody dilution buffer (Solarbio, Beijing, China) and replaced by phosphate buffer saline (PBS) as a blank control and Rabbit IgG Control (1:200 dilution; R&D Systems, USA) as a positive control. Then each section was rinsed by PBS for 10 min at room temperature to wash out unspecific signal. The EnVision System with diaminobenzidine (Dako Cytomation; Copenhagen, Denmark) was used as the chromogenic substrate to carry out secondary antibody staining for 30 min. Immunostaining results were evaluated in high-power fields (400 ×) using an Olympus CX41 microscope commencing from the first representative field on the left-hand side of the section. The presence of brown color was indicated as positive reactivity. Immunostaining for CD34 was only recorded as positive ( +) and negative (–). The endothelial cells stained with brown color, which were constituted vascular lumen in the form of cluster, were considered as positive. Bcl-2 staining was scored depending on the proportion of positively stained tumor cells, and the intensity of staining was assessed as negative (–), weak ( +), moderate (+ +) and intense (+ + +). Staining of ≤ 5% positive tumor cells was defined as negative (–). For positive immunoreactivity, staining of 5–10% positive tumor cells was defined as weak ( +), 10–30% was defined as moderate (+ +) and more than 30% was defined as intense (+ + +). All sections were assessed by two experienced pathologists blind to each patient’s clinical information. Where discrepancies arose, the section would be evaluated by the third pathologist. The staining of CD34 and Bcl-2 were shown in Figs 1 and 2.

### Statistical analysis

All statistical analyses were performed by SPSS software version 20.0 (IBM Corporation; Armonk, United States). Student's *t*-test or Pearson’s χ2 test was used to examine the relationship between the response to NACT and the patients’ clinicopathological parameters as appropriate. The correlation between CD34/Bcl-2 staining before NACT and the response to NACT was determined by Pearson’s χ2 test. The variation of CD34/Bcl-2 staining before and after NACT were compared using McNemar’s test or Wilcoxon signed-rank test. A threshold of *P* < 0.05 was considered to indicate the statistical difference.

## Results

### Patient’s clinicopathological characteristics

A total of 67 patients with cervical cancer were accorded with the included criteria. All patients were divided into NACT effective group (*n* = 48) and NACT ineffective group (*n* = 19) based on their response to NACT. Therein, CR rate was 23.88% (16/67), PR rate was 47.76% (32/67), SD rate was 23.88% (16/67), and PD rate was 4.48% (3/67). Table 1 showed the patients’ clinicopathological characteristics in NACT effective and ineffective groups. No statistically significant differences in patients’ age, BMI and FIGO stage were found between two groups (*p* = 0.703, *p* = 0.106 and *p* = 0.343, respectively).

**Table 1:** Demographic and clinical characteristics of the patients in the NACT effective and ineffective groups

Clinical variables	NACT effective (*n* = 48)	NACT ineffective (*n* = 19)	*P* value
Age (years)			
Mean ± SD	52.29±10.56	52.92±6.33	0.703^a^
Range	24–72	46–67	
BMI (kg/m^2^)			
Mean ± SD	20.51±3.26	21.24±3.41	0.106^a^
Range	17.62~25.14	18.14~25.36	
FIGO stage			
IB3	7	5	0.343^b^
IIA2	35	7	
IIB	6	7	

^a^Student's *t*-test; ^b^*χ*2 test; *NACT* neoadjuvant chemotherapy, *BMI* body mass index. *SD* standard deviation. *FIGO* international federation of gynecology and obstetrics. Data are the number of patients, unless indicated

### CD34 and Bcl-2 expression before NACT and correlation with NACT efficacy

The relationship between the expression of CD34 before NACT and patients’ response to NACT was investigated. Positive CD34 expression before NACT was seen in 70.83% of NACT effective patients, while only 31.58% patients in the ineffective group had a positive expression of CD34. The proportion of positive CD34 expression in NACT effective group was significantly higher than the ineffective group (*P* = 0.005, Table 2).

**Table 2:** Expression of CD34 or Bcl-2 before NACT in the NACT effective and ineffective group

Before NACT		No. of patients (%)		*P*-value
		NACT effective	NACT ineffective	
CD34	**–**	14^a^ (29.17%^b^)	13^a^ (68.42%^b^)	**0.005**^**c**^
	**+**	34^a^ (70.83%^b^)	6^a^ (31.58%^b^)	
Bcl-2	**–**	7^a^ (14.58%^b^)	8^a^ (42.11%^b^)	0.084^c^
	**+**	14^a^ (29.17%^b^)	4^a^ (21.05%^b^)	
	**++**	14^a^ (29.17%^b^)	5^a^ (26.31%^b^)	
	**+++**	13^a^ (27.08%^b^)	2^a^ (10.53%^b^)	

^a^Number of patients; ^b^percentage of patients in NACT effective or ineffective group; ^c^*χ*2 test; *NACT* neoadjuvant chemotherapy

There was no significant difference between two groups in Bcl-2 expression before NACT (*P* = 0.084, Table 2). Besides, the proportion of different Bcl-2 stating stages in NACT effective group were similar (29.17% of weak, 29.17% of moderate, 27.08% of intense, respectively).

### The down-regulated expression of CD34 and Bcl-2 after NACT in effective group

To explore the effect of NACT on CD34 and Bcl-2, we compared the expression of these two biomarkers before and after NACT respectively in two groups. In NACT effective group, more than half (82.35%) patients’ expression of CD34 turned positive to negative after NACT (*P* < 0.001, Table 3). Similarly, Bcl-2 expression of NACT effective group significantly decreased after NACT (*P* < 0.001, Table 4). Up to 39.58% of patients turned negative in Bcl-2 expression after NACT, and another 29.17% of patients’ expression was decreased. While in NACT ineffective group, no significant difference between CD34 or Bcl-2 expression were found before and after NACT (*P* = 0.453 and 0.317, respectively, Tables 3 and 4).

**Table 3:** Changes in the expression of CD34 before and after NACT in the NACT effective and ineffective group

			Expression of CD34 after NACT		*P* value
			**–**	**+**	
Expression of CD34 before NACT	Effective	**–**	13 (92.86%)	1 (7.14%)	**<0.001**^**a**^
		**+**	28 (82.35%)	6 (17.65%)	
	Ineffective	**–**	11 (84.62%)	2 (15.38%)	0.453^a^
		**+**	5 (83.33%)	1 (16.67%)	

Data in table were shown the number and percentage of patients’ CD34 expression

^a^McNemar’s test; NACT: neoadjuvant chemotherapy

**Table 4:** Changes in the expression of Bcl-2 before and after NACT in the NACT effective and ineffective group

			Expression of Bcl-2 after NACT				*P*-value
			**–**	**+**	**++**	**+++**	
Effective	Expression of CD34 before NACT	**–**	4 (57.14%)	3 (42.86%)	0	0	**< 0.001**^**a**^
		**+**	10 (71.43%)	2 (14.29%)	1 (7.14%)	1 (7.14%)	
		**++**	6 (42.86%)	6 (42.86%)	2 (14.28%)	0	
		**+++**	3 (23.08%)	6 (46.16%)	2 (15.38%)	2 (15.38%)	
Ineffective	Expression of CD34 before NACT	**–**	5 (62.50%)	3 (37.50%)	0	0	0.317^a^
		**+**	0	3 (75.00%)	0	1 (25.00%)	
		**++**	0	1 (20.00%)	4 (80.00%)	0	
		**+++**	0	0	1 (50.00%)	1 (50.00%)	

Data in table were shown the number of patients and percentage of patients’ Bcl-2 expression; ^a^ Wilcoxon signed-rank test; NACT: neoadjuvant chemotherapy

## Discussion

For cervical cancer, large local tumor lesions and positive parametria may lead to poor prognosis. NACT has been used for down-staging of tumor and inhibition of distant metastasis [12, 13]. NACT is not the recommended approach in NCCN (National Comprehensive Cancer Network) clinical guideline, because of the controversy over survival improvement compared with surgery alone [14, 15]. However, in European guidelines, NACT followed with radical hysterectomy is mentioned as a treatment option. It has been demonstrated that effective NACT before surgery could improve the operability and radical curability [16, 17]. However, a proportion of tumors do not response or later develop resistance to NACT, possibly leading to worse prognosis and ultimately patient mortality. Hence, the present study was conducted to explore reliable predictive markers for the efficacy of NACT in patients with cervical cancer. These may be helpful for clinicians to screen suitable patients for NACT. We found that positive CD34 expression was significantly associated with effective NACT, while no significant difference was found in the pre-NACT expression of Bcl-2 among different NACT efficacy groups. Moreover, both CD34 and Bcl-2 expression were significantly down-regulated after NACT in effective group, albeit to differing degrees.

Angiogenesis plays an important role in cancerous process, including progression, invasion and metastasis [18, 19]. The angiogenic degree of tumor is strongly associated with the aggressiveness and clinical outcome [20]. CD34 is a transmembrane glycoprotein expressed in a vascular endothelial cell that has been used as a direct marker to highlight the microvascular density [21]. An immunohistochemical study was shown that high CD34 expression was significantly associated with tumor size, tumor stage and lymph node metastasis in cervical cancer [22]. The present study revealed a positive relationship between the pre-NACT expression of CD34 and the efficacy of NACT in cervical cancer. Patients with high CD34 expression tended to be more sensitive to NACT. A retrospective analysis involving 55 cervical cancer patients found that the complete response rate of chemotherapy in patients with high microvascular density are 84.21%, which was significantly higher than the low-density group [23]. Similar results were observed in breast cancer [24, 25] and colorectal cancer [26]. High expression of CD34 signifies the presence of an anomalous vessel pattern characterized by high microvascular density. Chemotherapeutic drugs could fully infiltrate into the center of cancer through the rich vascular network and achieve better NACT efficacy [27]. We, therefore, suggest that CD34 expression is suited to be the predictive biomarker of NACT response in cervical cancer. Immunohistochemical staining of CD34 before NACT helps to develop individualized treatment of cervical cancer patients. The patient with positive CD34 expression may be sensitive to NACT and have a good response. NACT has the benefit of reducing tumor size, sterilizing parametria and eradicating the micro-metastatic disease [28]. Thereby, it gives the chance of operability to these chemo-sensitive patients. The CD34 staining before NACT also help the clinicians to screen out those chemotherapeutic non-responders. This may help to avoid delays in treatment and exempt the patients from unnecessary exposure to the toxicity of chemotherapy.

Apoptosis results from the activation of two different pathways: the extrinsic pathway by death receptor signaling and the intrinsic pathway via the mitochondria. Bcl-2 protein is located at intracellular membranes and plays a pivotal role in the mitochondrial pathway [29]. Previous study has reported that Bcl-2 expression was increased with the severity of cervical tissue lesions [30]. The 5-year survival rate in cervical cancer was also positively correlated with Bcl-2 expression [31]. Immunohistochemical Bcl-2 expression has been studied for its predictive value of chemotherapy for various cancer types with conflicting results. A multicenter retrospective analysis by Juliana Florinda et. al found that high Bcl-2 expression was associated with poor prognosis in patients with extrapulmonary neuroendocrine cancer treated with platinum-based chemotherapy [32]. Zhu et. al also found that that Bcl-2-negative status was an independent predictor of pathological complete response in breast cancer patients treated with paclitaxel and carboplatin neoadjuvant chemotherapy [9]. However, some trials had drawn different conclusions that positive Bcl-2 may predict a favorable chemotherapeutic effect. For example, Carla and colleagues found that positive expression of Bcl-2 had a significant predictive value for chemotherapy response in primary laryngeal/hypopharyngeal squamous cell cancer patients [33]. As for cervical cancer, less study evaluated the predictive value of Bcl-2 expression in NACT. Our study showed that no significant difference was found in pre-NACT Bcl-2 expression among different NACT efficacy. The earlier clinical study conducted among 247 bladder cancer patients with NACT reported similar findings to ours [8]. With regard to cervical cancer, Harima et al. [34] also presented that the Bcl-2 expression prior to radiotherapy did not correlate with treatment response. Although Bcl-2 are closely related to the stage and survival of cervical cancer, its expression before NACT is not a predictor of NACT in cervical cancer.

Disturbing tumor vasculature and promoting tumor cell apoptosis have been the primary strategies in cancer chemotherapy. Exploring the variation of CD34 and Bcl-2 expression after the administration of NACT, our study found that there was a significant decrease in these two biomarkers among NACT effective patients. Previous study has demonstrated that the effective chemotherapy of prostatic cancer may shrink the tumor size by impairing the vascularization of tumor [35]. Our data is in agreement with the observation of Jong-Yun Woo et. al, which found that the microvascular density within glioblastoma was reduced after effective chemotherapy [36]. Gine’s Luengo-Gil et al. [37] also proved that decreased microvascular density was associated with tumor downstaging in breast cancer after chemotherapy. Thus, the down-regulated expression of CD34 and Bcl-2 may reflect an efficient NACT response in cervical cancer. Li and colleagues found that chemotherapy-mediated miR29b functions as a tumor suppressor on proliferation and angiogenesis of cervical cancer [38]. Nevertheless, further investigations are needed to identify chemotherapeutic molecular mechanisms.

## Conclusion

Our study has uncovered that positive CD34 expression before NACT are significantly associated with effective NACT in cervical cancer. We presented that CD34 may be the predictive biomarker of NACT efficacy, while the pre-NACT expression of Bcl-2 is not an independent predictor. The expression of these two indicators significantly decreases after NACT, which may indicate effective NACT. In the future, treatment and diagnosis based on the CD34 and Bcl-2 expression in NACT may yet comprise a promising strategy. However, further researches are still required to investigate the roles of CD34 and Bcl-2 in NACT for patients with cervical cancer.
